# Eudragit^®^ L100/Polyvinyl Alcohol Nanoparticles Impregnated Mucoadhesive Films as Ocular Inserts for Controlled Delivery of Erythromycin: Development, Characterization and In Vivo Evaluation

**DOI:** 10.3390/biomedicines10081917

**Published:** 2022-08-08

**Authors:** Shahla Mirzaeei, Shiva Taghe, Raid G. Alany, Ali Nokhodchi

**Affiliations:** 1 Nano Drug Delivery Research Center, School of Pharmacy, Kermanshah University of Medical Sciences, Kermanshah 6715847141, Iran; 2 Pharmaceutical Sciences Research Center, School of Pharmacy, Kermanshah University of Medical Sciences, Kermanshah 6715847141, Iran; 3 Student Research Committee, School of Pharmacy, Kermanshah University of Medical Sciences, Kermanshah 6715847141, Iran; 4 Drug Discovery, Delivery and Patient Care Theme, Faculty of Science, Engineering and Computing, Kingston University London, Penrhyn Road, Kingston upon Thames KT1 2EE, UK; 5 School of Pharmacy, Faculty of Medical and Health Sciences, The University of Auckland, Private Bag 92019, Auckland 1142, New Zealand; 6 Pharmaceutics Research Laboratory, School of Life Sciences, University of Sussex, Brighton BN1 9QJ, UK

**Keywords:** controlled-release, erythromycin, Eudragit^®^ L100, mucoadhesive films, nanoparticles, ocular drug delivery

## Abstract

The fast elimination of drugs from the cornea is one of many challenges associated with the topical administration of conventional dosage forms. The present manuscript aimed to prepare modified-release inserts containing erythromycin (ERY) to enhance drug delivery and address the aforementioned limitation. Film formulations were developed using Eudragit^®^ L100 (EUD) and Polyvinyl Alcohol (PVA) polymers. ERY-loaded EUD-based nanoparticles were developed by the colloidal dispersion method using PVA as the emulsifier. The film-casting method was applied to form the mucoadhesive films using sodium alginate, gelatin, cyclodextrin-α, and β as polymeric film matrices. Different physicochemical properties of the optimized formulations and in vitro release profiles were evaluated. The in vivo evaluation was performed by collecting tear samples of rabbits using a novel, non-invasive method following the administration of inserts in the cul-de-sac. The ERY amount was assayed using a microbiological assay. The developed films showed prolonged in vitro and in vivo release profiles over five to six days; they had suitable physicochemical properties and a tensile strength of 2–3 MPa. All formulations exhibited antibacterial efficacy against *E. coli* and *S. aureus* with more than 20 mm diameter of inhibited growth zones. None of the formulations caused irritation to the rabbit’s eye. The inserts showed promising pharmacokinetics with AUC_0–120_ of 30,000–36,000 µg·h/mL, a C_max_ of more than 1800 µg/mL at 4 h, and maintained drug concentration over the threshold of 5 µg/mL during the following 120 h of study. Nanoparticle-containing, mucoadhesive films could be fabricated as ocular inserts and can prolong the topical ocular delivery of ERY.

## 1. Introduction

Novel drug delivery systems (NDDS) such as vesicles, nanoemulsions, nanofibers, and nanoparticles have been shown to act as efficient carriers for the topical ocular administration of different drugs [1,2]. These systems are capable of overcoming many challenges and limitations associated with the administration of conventional topical eye drops. Poor drug ocular bioavailability is characteristic of simple aqueous solution eye drops where rapid and extensive drug loss happens due to tear dilution, blinking, nasolacrimal drainage, pulse entry, metabolism, and non-specific absorption [3,4]. It has been shown that NDDSs may enhance the water-solubility of lipophilic drugs and prolong the amount of their time on the ocular surface by virtue of their mucoadhesive nature and ability to promote sustained drug release [5,6,7]. Previous studies have demonstrated the efficacy and benefits of these systems for ocular delivery of antibacterial, antifungal, and anti-inflammatory agents [8,9,10].

Polymeric nanoparticles (PNPs) that are classified as nanocapsules and nanospheres have attracted the attention of researchers in the past few decades [11]. These systems are drug-containing polymeric matrices with particle sizes ranging from 10 to 1000 nm [11]. PNPs have a wide range of applications in various fields, especially in drug delivery [12]. PNPs have the advantages of promoting drug controlled release, prolonging residence time, targeting the drug to the site of action, and consequently reducing side effects and enhancing drug absorption [13]. In addition to these advantages, PNPs can improve the bioavailability of drugs in the eye without causing blurred vision upon ocular administration. They can overcome the ocular barriers and improve the penetration of the drug through the cornea [14,15]. Mucoadhesive films can prolong the drug residence time on the surface of the eye, resulting in enhanced ocular bioavailability. By loading the mucoadhesive films with nanoparticles, the advantages of both of these systems can be realized [16,17,18].

Erythromycin (ERY) is a macrolide antibiotic that has an antibacterial effect on both gram-positive and some gram-negative bacteria [19]. The main mechanism behind ERY’s antibacterial effect is the inhibition of protein synthesis by binding to the ribosomal peptidyl transferase of bacteria [20]. It was reported that ERY topical ocular ointment could prevent eye disease caused by methicillin-resistant *Staphylococcus aureus* [21,22]. Despite the wide-spectrum antibacterial effects, the conventional ERY ointment suffers poor patient compliance due to the need for frequent administration [23]. Hence, designing a prolonged-release delivery system is important to achieve a therapeutic level with less frequent administration.

A literature survey revealed that there are a limited number of studies aimed at developing an effective system for the improved ocular delivery of ERY. In a recent study, ERY-loaded nanostructured lipid carriers showed a prolonged release of 24 h with an enhanced corneal permeation [24]. In another study, a prolonged release of ERY (8 h release) was also observed from an in situ ocular gel formulation [25]. In a recent study, ocular inserts of ERY were fabricated and evaluated, and the results showed that the developed formulations can release ERY for 3 h [26]. To the best of our knowledge, the present study is the first to report on an ocular film prepared by a solvent-casting method that is intended for the ocular administration of ERY.

In the present study, mucoadhesive films loaded with nanoparticles were designed and developed using the biocompatible polymers Eudragit^®^ L100 (EUD) and polyvinyl alcohol (PVA) for topical ocular delivery of ERY. The optimized formulations were evaluated for different physicochemical and mechanical properties. The efficacy of formulations to achieve sustained drug release as well as the improved antibacterial effect was demonstrated with the aid of in vivo and in vitro studies.

## 2. Materials and Methods

### 2.1. Materials

Sodium alginate (ALG), cyclodextrin-α and β (CD-α and β), gelatin (GEL), PVA (*M*_w_ = 72,000) were purchased from Sigma Aldrich (St. Louis, MO, USA). EUD L100 was procured from Ropharma (Milan, Italy). Methanol, sodium hydroxide, and hydrophosphoric acid were procured from Merck (Darmstadt, Germany). ERY was supplied from Dr. Reddy’s Pharmaceutical Company, Bengaluru, India. Trypticase soy agar (TSA), thioglycollate broth, soybean casein digest agar, and sabouraud dextrose agar were purchased from Merck (Seoul, South Korea). The bacterial strains *Micrococcus luteus* (ATCC 4698), *Staphylococcus aureus* (ATCC 25923), and *Escherichia coli* (PTCC 1399) were procured from the American Type Culture Collection. All of the other compounds were of analytical grade and were used without further purification.

### 2.2. Preparation of ERY-Loaded Nanoparticles

EUD nanoparticles were prepared using the colloidal dispersion method and according to the method performed by Bodmeier et al., with some minor modifications [27]. Accordingly, specified amounts of EUD (10 mg/mL) and ERY (2 mg/mL) were dissolved in methanol and stirred (300 rpm) for 12 h at room temperature. The formed organic dispersion was then added dropwise to an aqueous solution of PVA in water (10 mg/mL), under magnetic stirring (800 rpm) until the nanoparticles were formed as a nanosuspension.

### 2.3. Preparation of Polymeric Ocular Inserts Containing ERY-Loaded Nanoparticles

**Figure 1:**
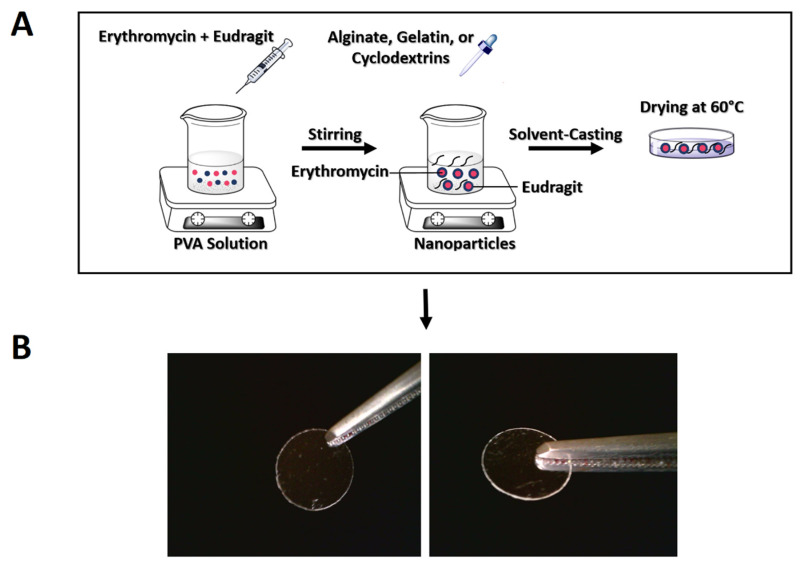
A schematic representation of the polymeric nanoparticle (PNP)-loaded film preparation procedure (**A**) visual appearance of nanoparticles-impregnated polymeric film insert (**B**).

Four different ocular inserts were prepared using CD-α, CD-β, ALG, and GEL polymers according to a previous study [28]. A predetermined amount of each polymer was dissolved in the appropriate solvent, under magnetic stirring at 50 rpm to obtain a clear 1% *w*/*v* solution. A specified volume (1 mL) of each solution was added dropwise to the EUD/PVA nanosuspension. The resulting mixtures were poured into Petri dishes and dried in an oven at 60 °C for 18 h, producing nanoparticle-loaded ocular films. The prepared films were cut into small pieces of predetermined dimensions (inserts) and stored under sterile conditions for further investigations (Figure 1). The whole procedure took place under aseptic conditions.

### 2.4. Characterization of ERY-Loaded EUD/PVA Nanoparticles

#### 2.4.1. Lyophilization

The prepared drug-loaded nanoparticles were lyophilized using a freeze-drier (Christ Freeze Dryer ALPHA 2-4, PLUS, Osterode, Germany) after being ultracentrifuged (Beckman-Coulter, Optima L-90K, Brea, CA, USA) at 30,000 rpm for 30 min at 4 °C [28]. Freezing took place at −85 °C for 4 h. The required time for vacuuming samples was 20 h at a negative pressure of 1.0 mbar. 

#### 2.4.2. Size and Zeta Potential Analysis

The size distribution and Zeta potential of nanoparticles were determined by a Zeta-sizer (Nano-ZS, Malvern, UK). Five milligrams of lyophilized samples were dispersed in 5 mL of distilled water under continuous stirring at 300 rpm for 5 min at room temperature. After complete dispersion of the nanoparticles, a turbid suspension was obtained and introduced to the zeta-sizer [28]. 

#### 2.4.3. Entrapment Efficacy

To evaluate the entrapment efficiency (*EE*), the dispersion containing drug-loaded nanoparticles was centrifuged at 30,000 rpm for 20–30 min to separate nanoparticles from the supernatant containing a non-entrapped drug [28]. The amount of entrapped ERY was calculated by subtracting the amount of the non-entrapped *ERY* (*W_free_*) that remained in the supernatant from the total amount of ERY used for the preparation of nanoparticles (*W_total ERY_*) divided by the total amount of drug (*W_total ERY_*) as formulated by Equation (1). The amount of non-entrapped drug in the supernatant was determined using a microbiological assay, which we explain in Section 2.9.

(1) $$EE\;\left(\%\right)=\frac{W_{total\;ERY}-W_{free\;ERY}}{W_{total\;ERY}}\times 100$$

#### 2.4.4. Drug Loading

The drug loading (*DL*) of nanoparticles was also determined according to the following equation, where *W_total ERY_* stands for the total amount of *ERY* used for the preparation of nanoparticles, *W_free ERY_* stands for the non-entrapped *ERY*, and *W_nanoparticles_* stands for the total weight of lyophilized nanoparticles [28]. The amount of non-entrapped drug in the supernatant was determined using the microbiological assay.

(2) $$DL\;\left(\%\right)=\frac{W_{total\;ERY}-W_{free\;ERY}}{W_{nanoparticles}}\times 100$$

### 2.5. Characterization of Ocular Inserts Containing ERY-Loaded Nanoparticles

#### 2.5.1. Thickness

The thickness uniformity is an important factor that should be considered in the evaluation of ocular inserts. The thickness was determined at six different points of each polymeric insert using a digital micrometer (Tork Craft Digital Micrometer, 0–25 mm, ME30025, Quanzhou, China) and the average was taken. 

#### 2.5.2. Folding Endurance

The prepared inserts were folded at the center repeatedly until they break or crack. The number of folding an insert could resist without breaking (folding endurance) was recorded. The test was repeated in triplicate and an average was taken. Folding endurance is a key factor in the determination of film flexibility [29].

#### 2.5.3. Percentage of Swelling

The swelling percentage of each ocular insert was determined by soaking them in a phosphate buffer (pH 7.4) at 37 °C. At the predetermined time intervals (30, 60, 90, and 120 min) inserts were weighed after the elimination of surface water using a filter paper, and based on the weight change through different times the swelling was calculated using Equation (3), where *W_t_* stands for the measured weight at time *t* and *W*_0_ is the initial weight of inserts [29]:

(3) $$Swelling\;\left(\%\right)=\frac{W_{t}-W_{0}}{W_{0}}\times 100$$

#### 2.5.4. Moisture Loss and Moisture Uptake

The moisture loss and uptake of ocular inserts were determined to ensure their stability and uniformity under dry and humid conditions [29]. These tests were carried out by putting a pre-weighed piece of polymeric film in a desiccator containing anhydrous calcium chloride (dry condition) and a saturated solution of aluminum chloride (humid condition) for three days. The final weight of each sample was then recorded and the percentages of moisture loss and uptake were determined by the formula described in Equation (4). The *W_f_* and *W*_0_ denote the final weight and the initial weight of the inserts, respectively.

(4) $$Moisture\;loss\;and\;uptake\;\left(\%\right)=\frac{\left|W_{f}-W_{0}\right|}{W_{0}}\times 100$$

#### 2.5.5. Surface pH

The surface pH of inserts was measured by placing each insert in a Petri dish containing 2% *w*/*v* agar in phosphate buffer (pH = 7.4) for 5 h under stirring conditions. The surface pH was measured using a pH meter (827 pH lab, Metrohm, Herisau, Switzerland). A mean of three readings was calculated.

#### 2.5.6. Tensile Strength

The tensile strength of the obtained inserts was studied using a universal mechanical testing machine (STM50, Santam, Tehran, Iran) [28]. Polymeric films with dimensions of 30 × 15 mm and a thickness of 1 mm were placed into the clamps and the stretching force was applied to samples to obtain tensile strength. Upon breaking, the amount of applied force and the elongation of films at break were recorded.

#### 2.5.7. Ex Vivo Mucoadhesion Time

A method adopted by Tofighia et al. [30] with slight modifications was utilized to determine the mucoadhesion time of formulations. There are also other similar methods to determine the mocuadhesive time [31,32]. Freshly excised sheep cornea was obtained from a slaughterhouse and attached by a two-sided glue tape to a microscopes slide. The slide was fixed to the basket of a disintegration apparatus filled with 900 mL PBS (pH = 7.4). The inserts were brought into contact with and pressed for 15 s onto the excised sheep cornea (hydrated with PBS). The disintegration apparatus was run where the fixed microscope slides moved upward and downward to come out and soak in the PBS repeatedly at a constant rate. The temperature was kept at 37 °C. The time required for the mounted inserts to completely detach from the cornea was recorded as the mucoadhesion time. The test was repeated three times for each sample.

### 2.6. Fourier Transform Infrared Spectroscopy (FTIR)

The FTIR analysis was performed to evaluate the drug-polymer compatibility and to detect any alteration of drug structure during preparation. FTIR spectra of ERY, PVA, EUD, drug-loaded ocular inserts and all polymers used for the preparation of inserts were generated by an FTIR spectrophotometer (IRprestige-21, Shimadzu Co., Tokyo, Japan) using the potassium bromide (KBr, 99.99%, Sigma-Aldrich, Burlington, MA, USA) pellets method [33]. The samples were ground with KBr and then were compressed into analytical pellets using a manual press under 10 tons pressure for 10 min. The spectra were recorded between 4000 to 400 cm^−1^.

### 2.7. Scanning Electron Microscopy (SEM)

The morphology of drug-loaded nanoparticles on the surface of polymeric films was observed by a scanning electron microscope (SEM TESCAN, MIRA3, Brno, Czech Republic). Samples were placed on the aluminum stub and were gold-coated prior to SEM imaging [33]. The accelerating voltage was set at 20 kV.

### 2.8. Antibacterial Efficacy

The inhibitory effect of prepared inserts on gram-positive and gram-negative bacteria was studied using *Staphylococcus aureus* (*S. aureus*) and *Escherichia coli* (*E. coli*), respectively, by the disk diffusion method [28]. Bacterial strains were cultured in tryptic soy broth (TBS) and incubated at 35 °C for 24 h, then diluted. Drug-loaded ocular inserts were cut into round disks of 6 mm diameter and placed onto TSA plates inoculated with bacterial suspensions, then incubated at 35 °C for 24 h. The zones of growth inhibition were observed and determined to evaluate the inhibitory effect of inserts.

### 2.9. Microbiological Assay 

According to previous reports, ultraviolet (UV) spectroscopy or high-performance liquid chromatography (HPLC)-UV methods are not suitable for the quantification of pure ERY [34,35,36]. Complicated derivatization is required in the case of drugs with weak UV absorption [37]. Also, according to USP, ERY assay is mainly performed by microbial assay, which has comparable accuracy to the HPLC method [34,35]. Therefore, this method was utilized in the present study. This method also has the advantage of a low limit of detection (LOD) [38]. As the disk diffusion method is used to perform the microbial assay, where a flexible disk is soaked with tear samples, there is therefore no need for invasive methods such as surgery, animal euthanasia or the sacrificing of animals [39,40].

The calibration curve of log_10_ of ERY concentration versus the diameter of the inhibition zone was constructed by microbiological assay [28]. A specific amount of ERY standard solutions in methanol (250, 125, 62.5, 31.25, 15.62, 7.81, and 3.90 µg/mL) was separately added to sterile blank disks and followed by drying the samples at room temperature. A McFarland standard suspension of *Micrococcus luteus* (*M. luteus*) was uniformly spread onto the TSA plates, then the disks were placed on the plates and incubated for 24 h at 35 °C. The diameter of inhibition zones was measured using a micrometer. All of the experiments were repeated three times and the mean ± SD at different concentrations were plotted to generate the calibration curve. The inter-day and intra-day accuracy of this method was determined and used as validation parameters.

### 2.10. In Vitro Release Study

To evaluate the release characteristics of ERY, a bi-chambered donor–receptor compartment model was assembled [28]. ERY ophthalmic ointment (Erythrolidine^®^, Sina Darou, Tehran, Iran), CD-α, CD-β, GEL, and ALG film inserts were subjected to in vitro release study. Eighty milligrams of each film insert formulation and 0.2 mg of ointment (along with 1 mL of PBS) were placed in a donor chamber that was separated from the receptor chamber (containing 24 mL PBS at pH of 7.4) using a dialysis membrane (Mw cutoff = 12,000–14,000 Daltons; Delchimica Scientific Glassware, Milan, Italy). At specific time intervals (1, 3, 5, 9, 14, 26, 38, 50, 74, 146 h), 100 μL samples were withdrawn and the whole receptor medium was replaced with fresh buffer (kept at the same temperature). The concentration of drugs retrieved from the receptor compartment at different time points was determined using the regression equation obtained by the microbiological assay method (described in Section 2.9). All the release experiments were repeated three times.

The release data were fitted to different kinetical models to predict the mechanism of drug release from inserts. The model with the highest correlation coefficient (R^2^) was selected as the best-fitted model.

### 2.11. Sterility Test

In order to maintain sterility, all ocular inserts were prepared under aseptic conditions. All of the prepared ocular inserts were exposed to UV light for 15 min and then were subjected to the sterility test. The sterility test was performed as per the USP guidelines. For this purpose, samples were placed for 28 days in three different culture media: (a) sodium thioglycolate broth to detect the growth of any anaerobic bacteria, (b) soybean-casein digest broth to observe aerobic bacterial contamination, and (c) sabouraud dextrose broth for determination of fungal growth. Positive and negative controls were used. 

### 2.12. In Vivo Ocular Irritation 

The Draize ocular irritation test was carried out on four male New Zealand albino rabbits, weighing 3.5–4 kg according to the method utilized by Gagandeep et al. [41]. Each of the prepared inserts along with 100 μL sterile PBS was separately placed in the cul-de-sac of the right eyes of a rabbit. An equal volume of phosphate buffer was used as a control in the left eyes of rabbits. The symptoms of ocular irritation or damage to the cornea, iris, and conjunctivae including abnormal discharge, conjunctival redness, swelling, corneal opacity, etc. were observed and scored on a 3-grade scale (0 = no alteration, 1 = mild alteration, 3 = obvious alteration) [42]. The sum of the scores obtained for each category was calculated.

### 2.13. In Vivo Bioavailability Study

The in vivo release of ERY from ocular inserts was determined using male New Zealand albino rabbits weighing 3.5 to 4 kg [28]. The animals were kept under the same diet and conditions. Before the test, the eyes of rabbits (*n* = 30) were washed with phosphate buffer. Small circular pieces (Ø = 5–6 mm) of ocular inserts (25 mg) containing 1.265 mg of ERY with 100 μL buffer were inserted in the conjunctival sac of animals. The marketed ophthalmic ointment of ERY (Erythrolidine^®^, Sina Darou, Tehran, Iran) was also examined as a control. At specific time intervals, samples were taken from the tears of rabbits by a novel, non-invasive method without the requirement for any surgical intervention which can cause discomfort to animals. Briefly, sterile paper discs with a diameter of 5 mm were soaked for 10 s with tear samples of rabbits and then assayed with the microbiological disk diffusion method described in Section 2.9. 

### 2.14. Statistical Analysis 

In the present study, all the experiments were carried out in triplicate, and data were reported as mean ± SD. Statistical analyses were carried out using the paired T-test and Kruskal-Wallis tests. The statistical significance for all tests was confirmed at *p* < 0.05.

## 3. Results and Discussion

### 3.1. Characterization of ERY-Loaded EUD/PVA Nanoparticles

Colloidal dispersion methods are commonly used for producing nanoparticles aiming at high entrapment efficiency, stability, and low toxicity. In the present study, a nanoparticle formulation was prepared for ocular delivery of ERY, using EUD as a coating polymer in the presence of PVA as a dispersing agent. Different formulations were prepared by changing experimental variables including the concentration of polymers, concentration of polyvinyl alcohol as a dispersant agent, and the weight ratio of drug to polymers, and the best set of these variables was chosen as the optimized formulation. Accordingly, the optimized formulation was developed resulting from a solution of EUD (10 mg/mL) and ERY (2 mg/mL) at a PVA concentration of 10 mg/mL. The higher concentrations of polymers led to larger particle sizes and higher viscosity which can avoid the formation of smaller particles by increasing the surface tension [43]. The mean particle size obtained for the formulation was in an appropriate nano-range (66.44 ± 4.19 nm), and the size distribution was narrow enough (PDI = 0.40 ± 0.03), to have a uniform distribution. This small particle size has the advantage of the increased surface-to-volume ratio resulting in enhanced solubility and bioavailability of the drug. Furthermore, formulations with nano-range particle sizes are less likely to cause any irritation to the eye [4]. In addition, nanoparticles showed a negatively charged Zeta potential (−6.03 ± 2.34 mV), which could mainly be attributed to the negative charge of EUD [44]. To achieve a suitable *EE*%, an optimized viscosity of PVA solution should be preserved (10–20 mPa.s) to prevent solubilizing of the drug in the aqueous phase. The *EE*% of nanoparticle formulation was up to 50.71 ± 3.16%, showing promising entrapment of ERY in the nanoparticles [45] (Table 1).

### 3.2. Characterization of Ocular Inserts

Nanoparticles were incorporated into different polymeric films to form inserts suitable for the ocular delivery of ERY. Ocular inserts were produced from different polymers by the solvent-casting method [46]. All of the prepared polymeric films had uniform and homogenous surfaces and were sufficiently transparent with no visible cracks or imperfections, corroborating the efficiency of the method used for the preparation of inserts. An ALG-based ocular insert is shown in Figure 1B. 

The measured weight variation and thickness of different polymeric films are shown in Table 2. Accordingly, the GEL formulation was the heaviest, followed by CD-α films. ALG films were the lightest of all the prepared inserts. The sufficient thickness uniformity of all prepared films is another finding which can be concluded from the obtained data. The thinnest film was found to be that of ALG (0.148 ± 0.004 mm), while the greatest thickness belonged to GEL (0.172 ± 0.002 mm). The low standard deviation for different formulations indicates the acceptable uniformity of all prepared polymeric films.

The folding endurance was another important property examined in this study. This parameter indicates the ability of each film to withstand frequent folding, which is important for the production, shipping, and handling of the insert. According to the results, all the prepared films possessed appropriate folding endurance withstanding 227–355 folds (Table 2). As shown in Table 2, GEL was the most resistant/durable, starting to crack but not break after 355 times of folding. The results of folding endurance confirm the desirable film-forming capability of the processed polymers, especially gelatin, and indicates that these inserts can potentially be used as inserts with sufficient mechanical flexibility [47,48]. 

The weight variation for each polymeric film, due to swelling, during the 2 h study is presented in Table 2. The results indicate that throughout the study period, the swelling percentage of the drug-loaded GEL insert was greater than other formulations with an almost 200% increase in weight. This could be attributed to the higher hydrophilicity of GEL, thus a high capacity to absorb water by the polymer network. The swelling percentage is likely to affect drug release from the formulations. The least swelling percentage was demonstrated by ALG (less than 150%).

It was observed that uptake and loss of moisture were insignificant in all the formulations (all less than 3%). ALG and GEL films were found to gain the least and the highest amount of moisture, respectively. The highest and lowest percentage of moisture loss was attributed to the GEL insert and CD-α insert, respectively. These results indicate that polymeric films have an acceptable hindrance to moisture transfer and are likely to be stable in both dry and wet conditions [49].

Surface pH is one parameter that should be evaluated as part of the development of any topical ophthalmic products as it is likely to affect their biocompatibility. Generally, topical ophthalmic formulations may cause discomfort, and irritation and induce reflex tear production leading to faster drug elimination via nasolacrimal drainage. In this study, all the formulations possessed a pH (between 6.5 and 6.7) which can be well tolerated by the eyes and cause minimal irritation and discomfort [50,51].

The formulations were ranked from highest to lowest tensile strength as follows: GEL > CD-α > CD-β > ALG. The tensile strength of all the formulations was in the range of 2.10 ± 0.01 to 3.42 ± 0.01 MPa, corresponding to ALG and GEL films, respectively. In a similar study, the tensile strength of 1.7 MPa was considered suitable for ocular administration [52]. Furthermore, multiple studies reported tensile strength values in a range of 1–3 MPa for similar ocular inserts [53,54]. The importance of having a suitable tensile strength is that the ocular insert would resist fast erosion (pulse drug release and rapid elimination) as well as showing little or no discomfort as a result of eye movements [55].

The investigated formulations showed a mucoadhesion time of between 8.4 to 10.7 min that is significantly enhanced compared to conventional eye formulations that are eliminated from the eye immediately (within 60–90 s) after administration. Table 2 presents the mucoadhesion times for different formulations to the sheep cornea. The CD-α and CD-β inserts provided the longest mucoadhesion times due to their hydrophilic nature. GEL also showed prolonged mucoadhesion time to the cornea compared with ALG, which could be ascribed to the relatively higher hydrophobicity of ALG. It is anticipated that the developed formulations would show longer residence times in vivo.

### 3.3. Fourier Transform Infrared Spectroscopy (FTIR)

**Figure 2:**
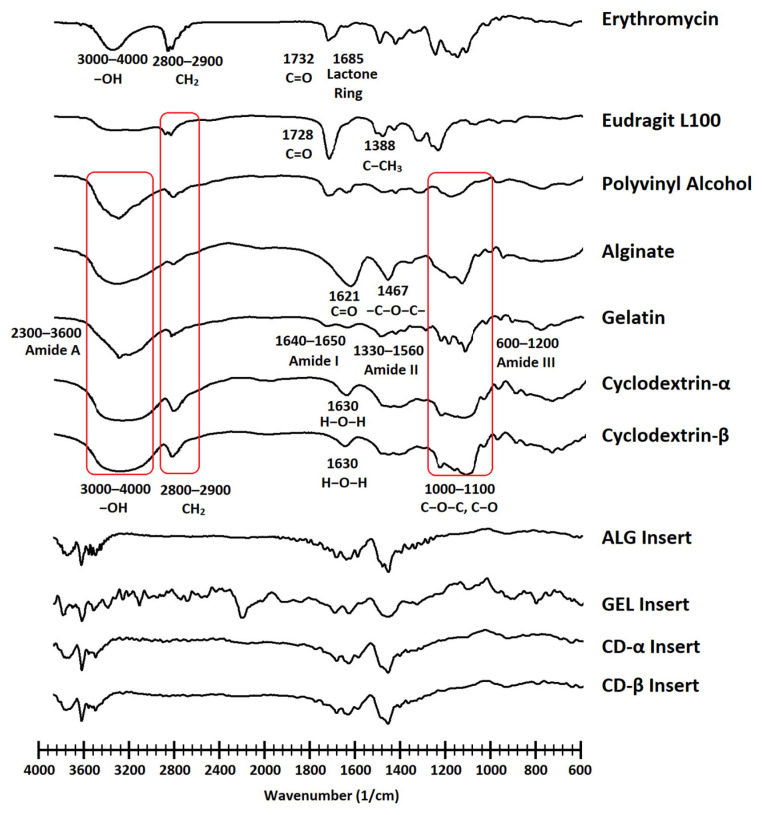
The FTIR spectra of erythromycin, polymers, and ocular inserts (ALG, GEL, CD-α, and CD-β inserts).

Figure 2 outlines the FTIR spectra of ERY, the polymers, and the ocular inserts. All formulations retain the characteristic peaks of ERY. The peak appeared around 3464 cm^−1^ and is assigned to the OH group of ERY. Peaks at around 1732 and 1685 cm^−1^ are attributed to ketone and lactone groups of ERY, respectively. CH_2_ bending of ERY also appeared between 1340–1460 cm^−1^. The characteristic peaks of EUD appeared around 1728 and 1388 cm^−1^, respectively corresponding to C = O vibration and CH_2_ stretching. Peaks at around 2800–2900 cm^−1^ representing the symmetrical and asymmetrical CH_2_ bond stretching are attributed to PVA, EUD, and matrices’ polymers. Peaks appeared at 1000–1100 cm^−1^, assigned to the C-O and C-O-C bond stretching of polymers. The FTIR spectrum obtained for each formulation represents the characteristic peaks of the polymers used in the preparation of the formulation along with the characteristic peaks of the drug. Based on the obtained results, it can be concluded that there was no interaction between the drug and the polymers and there is polymer-drug compatibility. It seems that a polymer-polymer interaction has occurred between the methacrylic acid functional group of EUD and GEL according to the obtained spectrum of GEL insert [56]. The appearance of new peaks in GEL inserts spectrum in the range of 1600–3400 cm^−1^ which is due to N-H stretching confirming the engagement of GEL amine groups in the abovementioned interaction.

### 3.4. Scanning Electron Microscopy (SEM)

**Figure 3:**
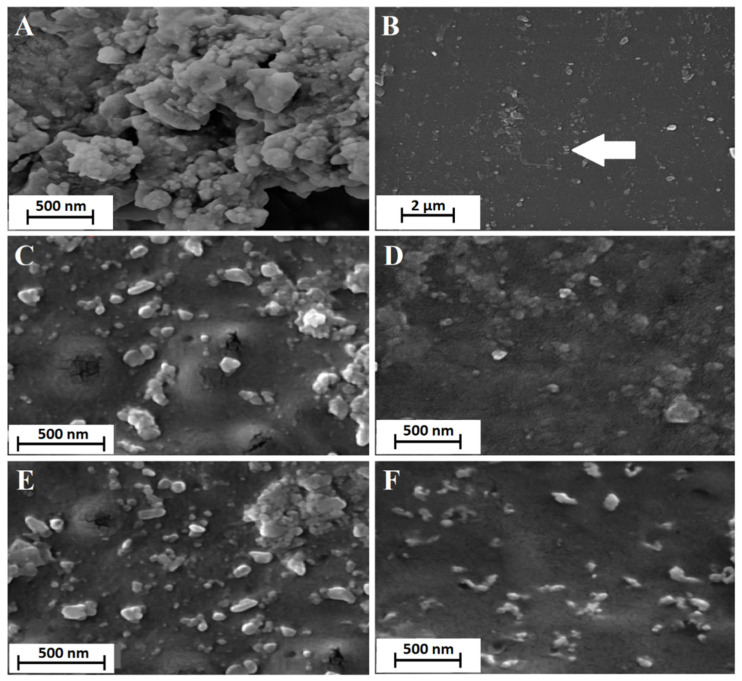
SEM images of erythromycin-loaded nanoparticles (**A**,**B**), CD-β insert (**C**), GEL insert (**D**), ALG insert (**E**), and CD-α insert (**F**).

Scanning electron micrographs revealed almost multi-dimensional, drug-loaded nanoparticles without aggregation (Figure 3). The nanoparticles possessed a smooth and uniform surface. As was previously reported, the loading of nanoparticles on the surface of polymeric films instead of distributing through the polymeric matrix could cause surface roughness [57]. The SEM photograph of polymeric films loaded with nanoparticles illustrates the uniform distribution of the nanoparticles in the polymeric matrices of the fabricated films. The uniform distribution of nanoparticles in the polymeric matrix of inserts was observed for all prepared formulations (Figure 3).

### 3.5. Antibacterial Efficacy

**Figure 4:**
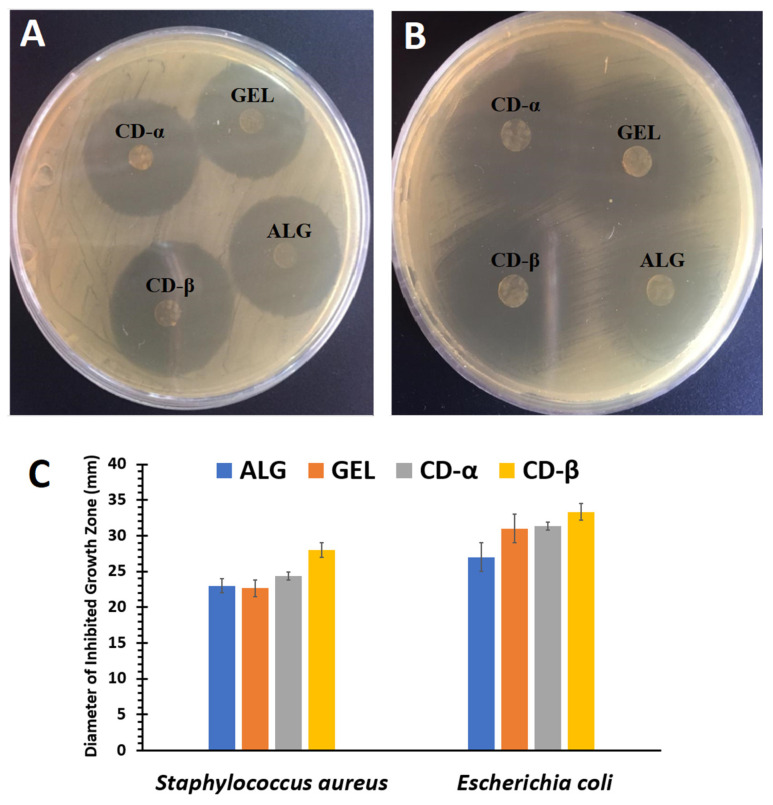
The antibacterial efficacy of ERY-loaded GEL, ALG, CD-α, and CD-β ocular inserts against *E. coli* (**A**) and *S. aureus* (**B**), and the comparative graph (**C**) of the diameter of inhibited growth zones (mm) obtained for inserts against these microorganisms (*n* = 3).

Figure 4 represents the antibacterial efficacy of ocular inserts against *S. aureus* and *E. coli*. The mean diameter of the inhibition zone in the presence of each prepared insert was determined and compared. At first glance, the inhibition zone could be observed in the vicinity of all the drug-loaded inserts against both gram-positive and gram-negative bacterial strains (Figure 4). Based on the previous reports, ERY has an antibacterial efficacy against both gram-positive and gram-negative bacteria [58,59]. From Figure 4, it is obvious that the antibacterial efficacy of ERY was preserved in the ocular inserts. However, no inhibitory activity could be observed in the plates containing blank inserts. The diameter of the inhibition zone was found to be larger in the presence of *E. coli*, in comparison with *S. aureus*, indicating the better antibacterial potential of ocular inserts against gram-negative bacteria. 

### 3.6. Microbiological Assay

The microbiological assay against *M. luteus* was carried out to construct the calibration curve of ERY in the concentration range of 3.90–250.00 μg/mL. The calibration curve followed the regression equation of 1.2528x + 0.0229 (R^2^ = 0.9690); where Y is the mean diameter of the zone of inhibition and X is the concentration of ERY. The inter-day and intra-day accuracy and precision of the method were evaluated at three different concentrations of the drug (Table 3). The results corroborated the acceptable accuracy and precision of the method for the determination of drug concentration through the study.

### 3.7. In Vitro Release Study

**Figure 5:**
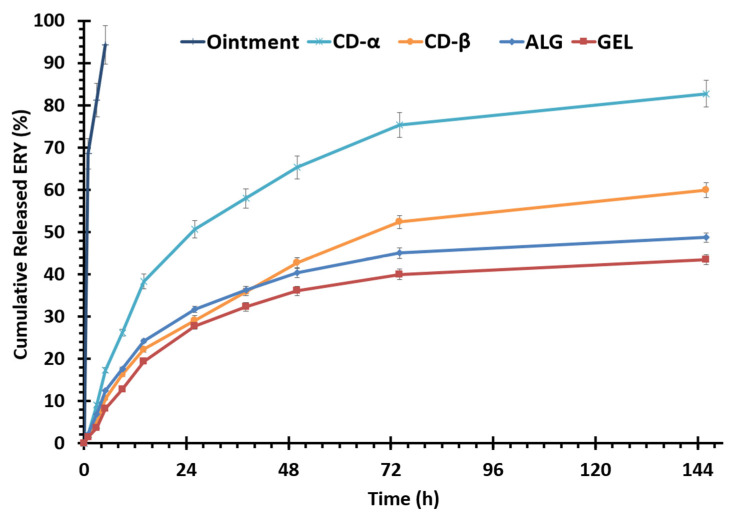
Cumulative release of erythromycin (expressed as a percentage) vs time profiles for the marketed ophthalmic ointment of erythromycin, GEL, ALG, CD-α, and CD-β ocular inserts in PBS (pH = 7.4) at 37 °C.

The in vitro release profile of ERY from EUD/PVA nanoparticles, loaded in different ocular inserts along with the nanosuspension, is illustrated in Figure 5. The most striking feature of the developed formulations was the sustained release pattern of the drug over more than 140 h compared to the ointment formulation, which released more than 90% of its drug content during the first 5 h. It seems that despite the hydrophobic nature of the base of this ointment, which is white petrolatum, the formulation was not able to release the drug in a controlled manner. The reason behind this rapid release is that the drug is suspended and not dissolved in the ointment base as it is highly water-soluble; hence, right after placement in the test apparatus at the temperature of 37 °C, the ointment base melted and the drug was rapidly released into the receptor medium. All the insert formulations potentially released 40–80% of their drug content over a prolonged duration (Figure 5). Through the first 12 h, all insert formulations released between approximately 15–35% of their drug content which was followed by a slow-release phase. From the release profiles presented in Figure 5, it can be seen that amongst the investigated inserts, CD-α, CD-β, and ALG showed the most promising release profile by releasing 82.76 ± 3.22%, 59.85 ± 1.75%, and 48.69 ± 1.12% of the drug in 146 h.

The release profile clearly shows a controlled release process of ERY. The steeper slope of the release profile during the initial hours of the study has likely occurred due to the fast release of the drug loaded onto or near the surface of nanoparticles, which is followed by a sustained-release of the ERY distributed in the polymeric matrix. The CD-α and CD-β ocular inserts showed higher percentages of the released drug due to the higher water solubility of matrix polymers and higher swelling percentage compared to the ALG insert [60]. Despite the higher degree of swelling compared to ALG, GEL indicated the lowest amount of released drug probably due to the interaction that occurred between GEL and EUD that led to a semi-rigid structure that inhibits the drug release [56]. A similar result was reported by Cetin et al., who designed and evaluated the diclofenac sodium-loaded EUD nanoparticles [44]. A 55-day release of the antifungal drug itraconazole was reported in a similar study [29].

Table 4 represents the R2 values obtained by fitting release data to different kinetical models. All formulations followed the Higuchi kinetical model. This indicated that the formulations released the drug mainly by the diffusion phenomenon. Accordingly, during the initial hours, the surface-loaded drug was released at a more rapid rate; the dissolution medium was then diffused into the inserts and dissolved the drug at a sustained rate.

### 3.8. Sterility Test

After sterilization of ocular inserts by UV irradiation, the growth of microorganisms in the presence of each ocular film containing blank nanoparticles was examined. Different groups including a positive control, negative control, and test groups were visually examined. No significant change was observed in the turbidity of media for the negative control and test tubes, while in the positive control the growth of microorganisms was detected. According to these results, it can be concluded that the whole procedure was aseptic, the ocular inserts were sterile, and that they could be confidently used for in vivo study.

### 3.9. Irritation Test

**Figure 6:**
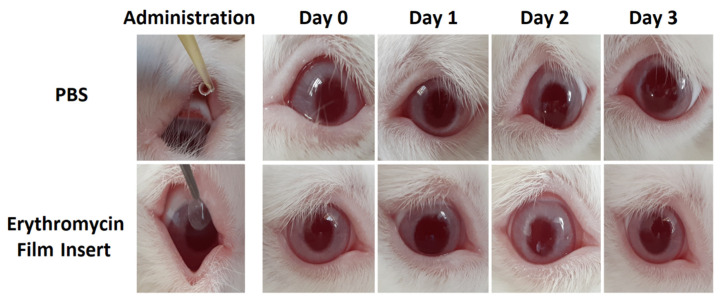
Images of rabbits’ eyes treated with phosphate buffer solution (PBS) and ALG ocular insert during 3 days of examination by Draize test.

The possibility of inducing irritation after the insertion of ocular inserts in the eyes of the rabbit was examined by the Draize test to ensure that the prepared inserts can be potentially used by humans without causing pain or discomfort. Upon placing ocular inserts in the eyes of rabbits, at specific time intervals (0 h, 24 h, 48 h, and 72 h) the eyes were carefully inspected. The obtained scores are classified in Table 5. Also, Figure 6 indicates the result of the irritation test for one of the ocular inserts and control groups. Irritancy scores of GEL, CD-α, and CD-β were observed to be 0 within three days. There was no sign of irritation, redness, swelling, corneal opacity, intensive tearing, frequent blinking, or any other visible abnormality for any of the formulations except for ALG, which showed a mild redness during the first day of administration. The appearance was similar to control eyes. As for the fact that rabbits possess eyes that are more vulnerable to damage and irritation than humans, it could be generalized that such formulations can be inserted into human eyes without causing any significant irritation or abnormalities [61].

### 3.10. In Vivo Tear Fluid Bioavailability Study

Taking the in vitro release results into consideration, all formulations (CD-α, CD-β, GEL, and ALG) have been subjected to in vivo study. In this study, sampling from the animal tears was done by a novel method that is more animal-friendly and has been set up for the first time by our team in a previous study [28], hence a possible alternative to using common invasive sampling methods. In previous investigations, in vivo sampling required anesthetizing or sacrificing animals [62]; however, in this study sampling was performed by collecting the rabbits’ tears using a sterile paper disk which was then assayed by the microbial assay for ERY content. To the best of our knowledge, no previous studies have reported on a similar method of in vivo sampling.

**Figure 7:**
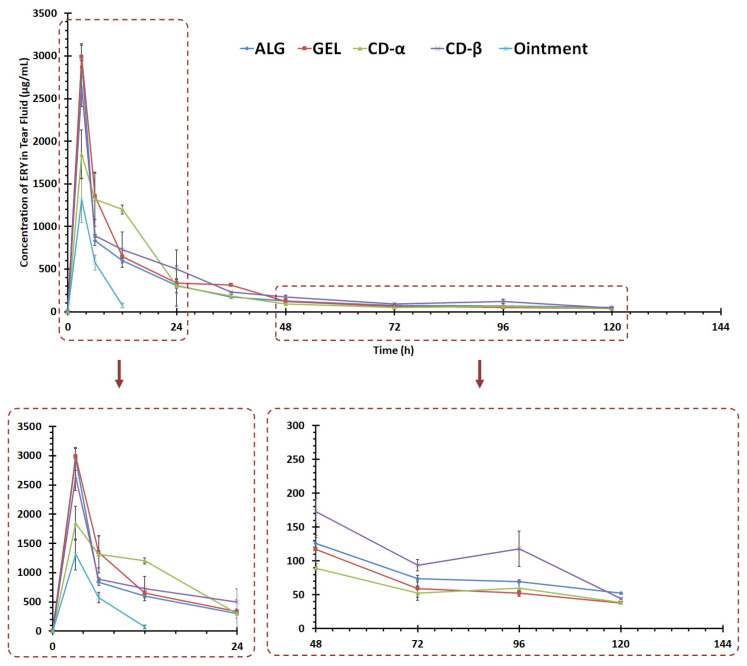
ERY concentration in tear fluid following administration of marketed ophthalmic ointment of erythromycin, ALG, GEL, CD-α, and CD-β ocular inserts (*n* = 30).

According to Figure 7, a sharp peak appeared in the tear fluid release profile of ERY described as C_max_ for all formulations at the initial hours reaching 2990.35 ± 133.20, 2945.43 ± 196.72, 1845.21 ± 286.60, and 2640.18 ± 234.97 μg/mL for GEL, ALG, CD-α, and CD-β insets, respectively. Meanwhile, the marketed formulation could reach a C_max_ of 1309.91 ± 260.90 μg/mL, which is significantly lower than the film inserts. The sharp peak indicates the burst release of ERY from each formulation, which could be considered beneficial. This high amount of released drug can cause an initial shock against microorganisms. As has been proven in previous studies, topical formulations have the drawback of poor absorption and precorneal loss, which leads to the fact that only about 1–10% of the drug can be absorbed intraocularly [63]. The developed inserts can beneficially prolong the residence time of the drug on the ocular surface four times longer than the marketed formulation. The ophthalmic ointment was washed out of the conjunctival sac within 5 h of investigation, and after this interval, the drug was not detectable in the tear fluid. Also, as mentioned earlier, due to high water solubility, ERY is not soluble in the ointment base and is washed out of the eye right after administration and the dissolving of the ointment in the conjunctival sac. 

Increasing the ocular-surface (tear fluid) availability of ERY is likely to increase the intervals needed between drug administrations, thus overcoming the requirement for frequent use of the drug where fewer side effects are expected. This is likely to improve patient compliance due to less frequent administration. Another positive aspect of the studied formulations is that the tear concentration of ERY is beyond the MIC against microorganisms over 120 h of in vivo analysis. The most AUC_0–120_ was measured for CD-β,followed by GEL, CD- α, and ALG, respectively. The pharmacokinetic parameters of the developed insert are indexed in Table 6.

In a similar investigation, a nanoparticles impregnated ocular insert containing acetazolamide was prepared. The data obtained from in vivo studies indicated an enhanced therapeutic effect of nanoparticle formulation compared to a conventional solution [57]. As reported by Chhonker et al., amphotericin B-loaded nanoparticles showed an enhanced pharmacokinetic effect compared to the marketed formulation [64]. Taghe et al. have reported the same sustained-release profile of azithromycin for ocular administration from mucoadhesive films impregnated with nanoparticles [28]. In a similar study, EUD-based nanofibrous inserts indicated the sustained release of ofloxacin with a tear concentration above the MIC for 96 h [65].

## 4. Conclusions

Four different mucoadhesive polymeric film formulations containing ERY-loaded EUD/PVA nanoparticles were designed and developed as ocular inserts to overcome the challenges related to the administration of conventional eye formulations including limited bioavailability, short residence time, and the requirement of frequent administration. The developed nanoparticles possessed suitable particle size and over 50% entrapment efficacy. All ocular inserts indicated strength, flexibility, and proper mechanical properties to be used on the ocular surface without undesirable effects. No interaction between drug and polymers was observed and morphological examination indicated a uniform surface with a good distribution of nanoparticles in the polymeric matrices. All formulations showed antimicrobial efficacy against *E. coli* and *S. aureus*. The in vitro study showed a controlled release profile extending over six days. CD-α and CD-β inserts showed the most promising in vitro release profiles by releasing 60–80% of the drug in 144 h. A novel non-invasive technique was established for in vivo tear sampling from rabbits. The microbiological assay showed 5 days of ERY release in the tear fluid for the observed formulations. Although all formulations showed promising pharmacokinetics to be used as ocular inserts, the formulation prepared by CD-β as the matrix component showed higher mucoadhesion, AUC_0–120_ and MRT among formulations, and hence can be selected as the optimized formulation. The in vivo testing test showed no significant signs of ocular irritation. Finally, it could be concluded that the ERY-loaded EUD/PVA nanoparticles incorporated into ocular films can be considered a promising ocular formulation for the extended-release of ERY. Patient compliance can be potentially enhanced using the developed inserts, where there is less need for frequent administration compared to the conventional ointment. The authors believed that these formulations have the potential to be further developed and evaluated for delivery of various topical ocular agents such as antibiotics, antifungals, ocular hypotensive, steroids and nonsteroidal anti-inflammatory, antihistamines, decongestants etc., but to prove their usefulness for a particular clinical indication systematic study should be performed.

## Figures and Tables

**Table 1:** Particle characteristics, drug entrapment, and loading of developed nanoparticle formulation.

Formulation	Particle Size (nm)	Polydispersity Index	Zeta Potential (mV)	*DL* (%)	*EE* (%)
Nanoparticles	66.44 ± 4.19	0.40 ± 0.03	−6.03 ± 2.34	22.41 ± 2.55	50.71 ± 3.16

Abbreviations: *DL* (Drug Loading), *EE* (Entrapment Efficiency).

**Table 2:** The physicochemical-mechanical characteristic of developed inserts.

Parameter		Formulations			
		ALG	GEL	CD-α	CD-β
Weight (mg)		20.19 ± 0.65	23.61 ± 0.25	22.14 ± 0.20	21.63 ± 0.30
Thickness (mm)		0.148 ± 0.004	0.172 ± 0.002	0.170 ± 0.006	0.155 ± 0.003
Folding endurance (times)		227.0 ± 5.2	355.3 ± 6.1	341.0 ± 8.5	303.1 ± 8.1
pH		6.64 ± 0.03	6.69 ± 0.02	6.50 ± 0.02	6.52 ± 0.04
Tensile Strength (MPa)		2.10 ± 0.01	3.42 ± 0.01	2.35 ± 0.07	2.30 ± 0.01
Swelling (%)	30 min	55.3 ± 0.8	101.0 ± 1.9	74.3 ± 0.4	80.3 ± 1.9
	60 min	91.2 ± 0.8	189.0 ± 3.6	154.0 ± 3.6	175.0 ± 0.4
	120 min	147.3 ± 0.8	194.0 ± 1.9	174.3 ± 1.9	181.0 ± 0.4
Moisture uptake (%)		0.77 ± 0.07	2.08 ± 0.09	1.62 ± 0.08	0.88 ± 0.13
Moisture loss (%)		1.16 ± 0.05	1.60 ± 0.07	0.51 ± 0.08	0.97 ± 0.13
Mucoadhesion time (min)		8.4 ± 0.4	9.2 ± 0.2	9.4 ± 0.3	10.7 ± 0.5

**Table 3:** The intra- and inter-day accuracy and precision for the microbial assay method measuring three different inhibitory concentrations of erythromycin against *M. luteus*.

Concentration of ERY (μg/mL)	Accuracy (%)		Precision (CV %)	
	Intra-Day	Inter-Day	Intra-Day	Inter-Day
125	102.947	112.268	0.494	0.703
62.5	94.581	114.266	0.401	0.477
31.25	116.908	110.410	0.418	0.520

Abbreviaitions: ERY (Erythromycin), CV (coefficient of variation).

**Table 4:** The correlation coefficients obtained by fitting the release data in different kinetical models.

Formulation	Correlation Coefficient (R^2^)			
	Zero-Order	First-Order	Higuchi	Korsemeyer-Peppas
ALG	0.9030	0.9341	0.9847	0.9606
GEL	0.9400	0.9595	0.9898	0.9572
CD-α	0.9096	0.9614	0.9858	0.9465
CD-β	0.9325	0.9579	0.9839	0.9837

**Table 5:** Ocular irritation test according to Draize test.

Formulation	Time (h)	Cornea		Iris	Conjunctivae			Total Score
		Opacity	Area of Cornea Involved	Interruption for Reaction to Light	Redness	Chemosis	Discharge	
Control	24	0	0	0	0	0	0	0
	48	0	0	0	0	0	0	0
	72	0	0	0	0	0	0	0
ALG	24	0	0	0	1	0	0	1
	48	0	0	0	0	0	0	0
	72	0	0	0	0	0	0	0
GEL	24	0	0	0	0	0	0	0
	48	0	0	0	0	0	0	0
	72	0	0	0	0	0	0	0
CD-α	24	0	0	0	0	0	0	0
	48	0	0	0	0	0	0	0
	72	0	0	0	0	0	0	0
CD-β	24	0	0	0	0	0	0	0
	48	0	0	0	0	0	0	0
	72	0	0	0	0	0	0	0

**Table 6:** The pharmacokinetic parameters of ERY-loaded ocular inserts tested in the rabbits’ eyes (*n* = 30).

Formulation	C_Max_ (μg/mL)	AUC_0–120_ (μg·h/mL)	MRT (h)
Ointment	1309.91 ± 260.90	4986.62 ± 630.85	3.67 ± 0.16
ALG	2945.43 ± 196.72	30,020.92 ± 45.54	24.85 ± 0.01
GEL	2990.35 ± 133.20	33,917.77 ± 3023.76	21.91 ± 0.10
CD-α	1845.21 ± 286.60	32,786.23 ± 3550.15	21.60 ± 0.48
CD-β	2640.18 ± 234.97	35,938.29 ± 602.54	28.27 ± 0.14

Abbreviations: C_Max_ (Maximum Concentration), AUC (Area Under the Curve), MRT (Mean Residence Time).

## Data Availability

Data will be sent to the applicants after sending their requests to the corresponding author. The applicants will need to sign a data access agreement.
